# Influence of azacycle donor moieties on the photovoltaic properties of benzo[*c*][1,2,5]thiadiazole based organic systems: a DFT study

**DOI:** 10.1038/s41598-023-41679-0

**Published:** 2023-09-05

**Authors:** Iqra Shafiq, Muhammad Khalid, Muhammad Adnan Asghar, Rabia Baby, Ataualpa A. C. Braga, Saad M. Alshehri, Sarfraz Ahmed

**Affiliations:** 1 Institute of Chemistry, Khwaja Fareed University of Engineering & Information Technology, Rahim Yar Khan, 64200 Pakistan; 2 Centre for Theoretical and Computational Research, Khwaja Fareed University of Engineering & Information Technology, Rahim Yar Khan, 64200 Pakistan; 3 Department of Chemistry, Division of Science and Technology, University of Education Lahore, Lahore, Pakistan; 4 Department of Education, Sukkur IBA University, Sukkur, 65200 Pakistan; 5 Departamento de Qu´ımica Fundamental, Instituto de Qu´ımica, Universidade de Sao˜ Paulo, Av. Prof. Lineu Prestes, 748, Sao Paulo, 05508-000 Brazil; 6 Department of Chemistry, College of Science, King Saud University, Riyadh, Saudi Arabia; 7 Wellman Center for Photomedicine, Harvard Medical School, Massachusetts General Hospital, Boston, MA 02114 USA

**Keywords:** Materials science, Optics and photonics

## Abstract

Fullerene free organic chromophores are widely utilized to improve the efficacy of photovoltaic materials. Herein, we designed D-π-A-π-D form chromophores (**TAZD1-TAZD5**) via end-capped redistribution of donor moieties by keeping the same π-bridge and central acceptor unit for organic solar cells (OSCs). To analyze the photovoltaic characteristics of these derivatives, DFT estimations were accomplished at B3LYP/6–311 G (d,p) functional. Different investigations like frontier molecular orbital (FMO), absorption spectra (UV–Vis), density of states (DOS), binding energy (E_b_), open circuit voltage (*V*_*oc*_), and transition density matrix (TDMs) were performed to examine the optical, photophysical and electronic characteristics of afore-mentioned chromophores. A suitable band gap (∆E = 2.723–2.659 eV) with larger bathochromic shift (*λ*_max_ = 554.218–543.261 nm in acetonitrile) was seen in **TAZD1-TAZD5**. An effective charge transference from donor to acceptor via spacer was observed by FMO analysis which further supported by DOS and TDM. Further, lower binding energy values also supported the higher exciton dissociation and greater CT in **TAZD1-TAZD5**. Among all the designed chromophores, **TAZD5** exhibited the narrowest *E*_gap_ (2.659 eV) and maximum red-shifted absorption in solvent as well as gas phase i.e. 554.218 nm and 533.219 nm, respectively which perhaps as a result of the phenothiazine-based donor group (**MPT**). In a nutshell, all the tailored chromophores can be considered as efficient compounds for promising OSCs with a good *V*_oc_ response, interestingly, **TAZD5** is found to be excellent chromophores as compared to all these designed compounds.

## Introduction

Solar energy has turn into a promising energy source which involve the phenomena of photoelectric effect and overcome the elevating power crisis as the sunlight is non-exhaustible, non-polluting and widely available^1^. Among promising and cost-effective substitutes for future sustainable energy are OSCs due to their exceptional advantages. OSCs have been proved as an effective scheme for light manipulation, which is capable of improving the light absorption process^2^. In this way, larger photocurrent is produced due to the light scattering. The photovoltaic innovation has gained consideration of academic along with industrial communities’ decades-long. The photovoltaic (PV) silicon-based solar cells were known as the foremost and popular energy gadgets owing to their remarkable eco-friendly nature, proficiency and low cost^3^. Crystalline silicon solar cells have the greatest manufacturing history with over 60 years of progress and their efficiency has increased to above 25% because of the improvements in their architecture^4,5^. However, they are fragile and possess non-tunable energy levels due to which the organic-based solar cells have become more popular in recent years. They possess various important characteristics such as; (i) light weight; (ii) low-cost materials; (iii) tunable energy levels; (iv) mechanical flexibility; (v) variety of structural modulations and (vi) compatibility with large manufacturing^6–9^. Furthermore, organic–inorganic perovskite photovoltaics have gained impressive power conversion efficiency (PCE) of 22.1% because of remarkable characteristics like intense absorption spectrum, greater charge mobility and long diffusion length of charges^10–12^. Another class of OSCs namely dye-sensitized solar cells (DSSC) has also captured significant attention owing to their stability besides tunable visual characteristics e.g. transparency and color^13–15^. Components in DSSCs, such as the dye catches special attention on because of light conversion capability to electricity supported by photoexcitation^13^. The DSSCs that are metal free are of great efficiency and advantageous because of their synthesis, purification and many optical properties by easy chemical modifications^16^. Although in beginning efforts to commercialize organic photovoltaics (OPVs) were difficult as a result of lower power conversion efficiency (PCE) than the approximated market viability of 15%^17^. Besides, the photovoltaic domain is developed with other very compelling substances like OSCs based on fullerene derivatives namely PC71BM, ICBA, and PC61BM. Organic solar cells (OSCs) keeping fascinating characteristics like simple processability, light weight, mechanical flexibility, high formulation area and ease made them significant alternative tools^18–21^. Due to the exceptional electronic and structural properties of fullerene-based OSCs, they have been widely examined since 1985^22^. Low reorganization energy of excitons^23,24^, elevated electron affinity^25^, and high mobility of electrons^26,27^ are some fascinating and distinctive properties of fullerene based OSCs. There also exhibit certain drawbacks in fullerene acceptors (FAs) which include less absorption in visible and near IR regions, poor photochemical and thermal stability^28^, non-tunable LUMO energies^29^ and less sunshine assimilation which impelled the researchers to search for some more strongly absorbing analogues^30^. Therefore, non-fullerene acceptors (NFAs) are utilized in the OSCs in place of FA owing to their flexible nature, higher fabrication area and wide tunability of their energy levels^31^. The non-fullerene small molecule acceptors (NF-SMAs) are regarded as remarkable constituents for proficient OSCs^32^. A rapid improvement in PCE is observed (~ 18–19%) in NFAs benefiting from years of research on fullerene-based BHJ materials^33,34^. The obvious increase in fill factor (FF) as well as short-circuit current (*J*_sc_) is also seen in NFA-based devices with greater open-circuit voltage (*V*_oc_) in comparison to their fullerene counterparts^35^. Literature is flooded with many examples in which fullerene free donor or acceptors are extensively utilized to improve the efficiencies of photovoltaic materials^36–39^. Keeping in view the importance of NF organic systems, herein, we have tried to design benzodithiophene based organic systems for high efficacy photovoltaic devices. For this purpose, we take a synthesized **X94FIC**^40^ fullerene free A-π-A-π-A architecture acceptor nature molecule and designed sequence of donor type D-π-A-π-D configured **TAZD1-TAZD5** chromophores by structural modification of end capped acceptors with efficient donor moieties. Perhaps, it is first ever systematic comparative study of impact of NF chromophores with five-, six- and seven-membered rings on the electrochemical as well as photophysical characteristics. To check the influence of donor groups on photovoltaic characteristics, DFT method was employed and has anticipated their significance in OSCs.

### Computational procedure

Gaussian 09 package^41^ was exploited to understand the photovoltaic response of benzodithiophene based organic (**TAZD1-TAZD5**). To choose the suitable functional for current investigation, a relative investigation of **X94FIC** *λ*_max_ outcomes among several TD-DFT functionals and experimental results was performed. For this purpose, the reference chromophore **X94FIC** was subjected to geometry optimization using four different functionals, including B3LYP^42^, M06^43^, MPW1PW91^44^ and ɷB97XD^45^ in acetonitrile solvent as range-separated functionals estimates HOMO–LUMO gaps and excited-state energies better^46–49^. Then these optimized geometries were applied to execute UV–Vis analysis in acetonitrile solvent and 818.864, 736.686, 676.142, and 495.461 nm values of *λ*_max_ were obtained at aforesaid functionals, respectively. The *λ*_max_ values of **X94FIC** obtained using these functionals were compared to the experimentally determined maximum absorption value of 783 *nm*^39^ of **X94FIC** chromophore. At B3LYP/6-311G (d,p) functional, closed harmony was seen with experimental results. Moreover, we also compared the band gap values of **X94FIC** calculated at aforesaid functional of TD-DFT (1.774, 2.142, 2.032 and 4.971 eV*,* respectively) with experimental ΔE value (1.41 eV)^39^ and interesting, good harmony with experimental results was seen at B3LYP, hence, this functional was selected for this study. First of all, structures of designed systems were optimized at B3LYP/6-311G(d,p) to get true minima geometries in acetonitrile solvent. The absence of any imaginary frequency specified that structures were at true minima potential energy surface. After the successful optimization of geometries, different analyses; FMOs, DOS, UV–Vis, *V*_oc_, E_b_ and TDMs were attained to inspect the optical, electronic and photophysical characteristics of afore-mentioned chromophores at B3LYP/6-311G (d,p) level of DFT/TDDFT in acetonitrile solvent. Nevertheless, in order to understand the effect of different media on UV–Vis properties, we performed absorption analysis in gas and acetonitrile at foresaid functional. For the extraction of data from output files, Gauss View 5.0 program^50^, Avogadro^51^, Chemcraft^52^, PyMOlyze 2.0^53^, and Origin^54^ software were utilized and the data was recorded in the form of graphs and tables.

## Results and discussion

**Figure 1:**
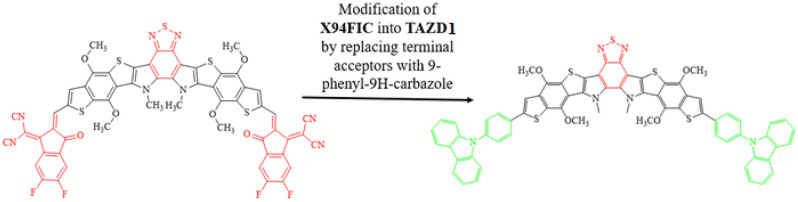
Modification of **X94FIC** into **TAZD1** by replacing terminal acceptors with four ring azacycle donor unit, drawn utilizing ChemDraw software (https://chemistrydocs.com/chemdraw-pro-8-0/).

**Figure 2:**
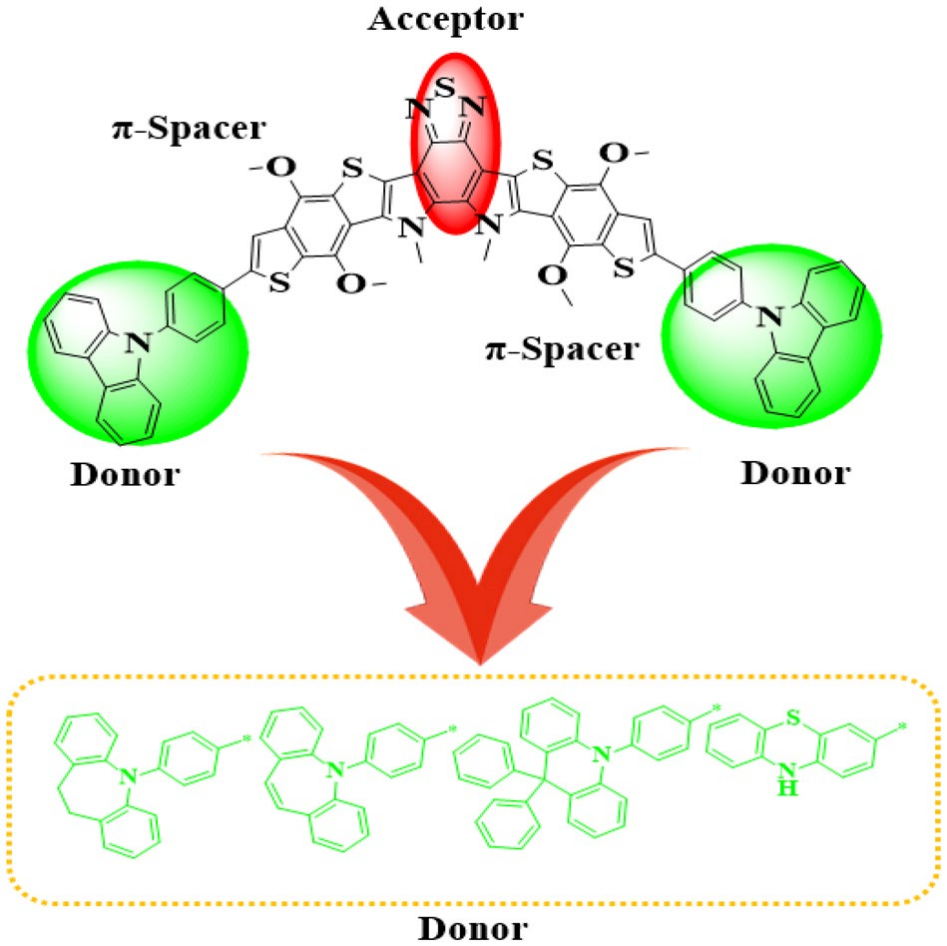
The sketch map of the **TAZD1-TAZD5** compounds.

**Figure 3:**
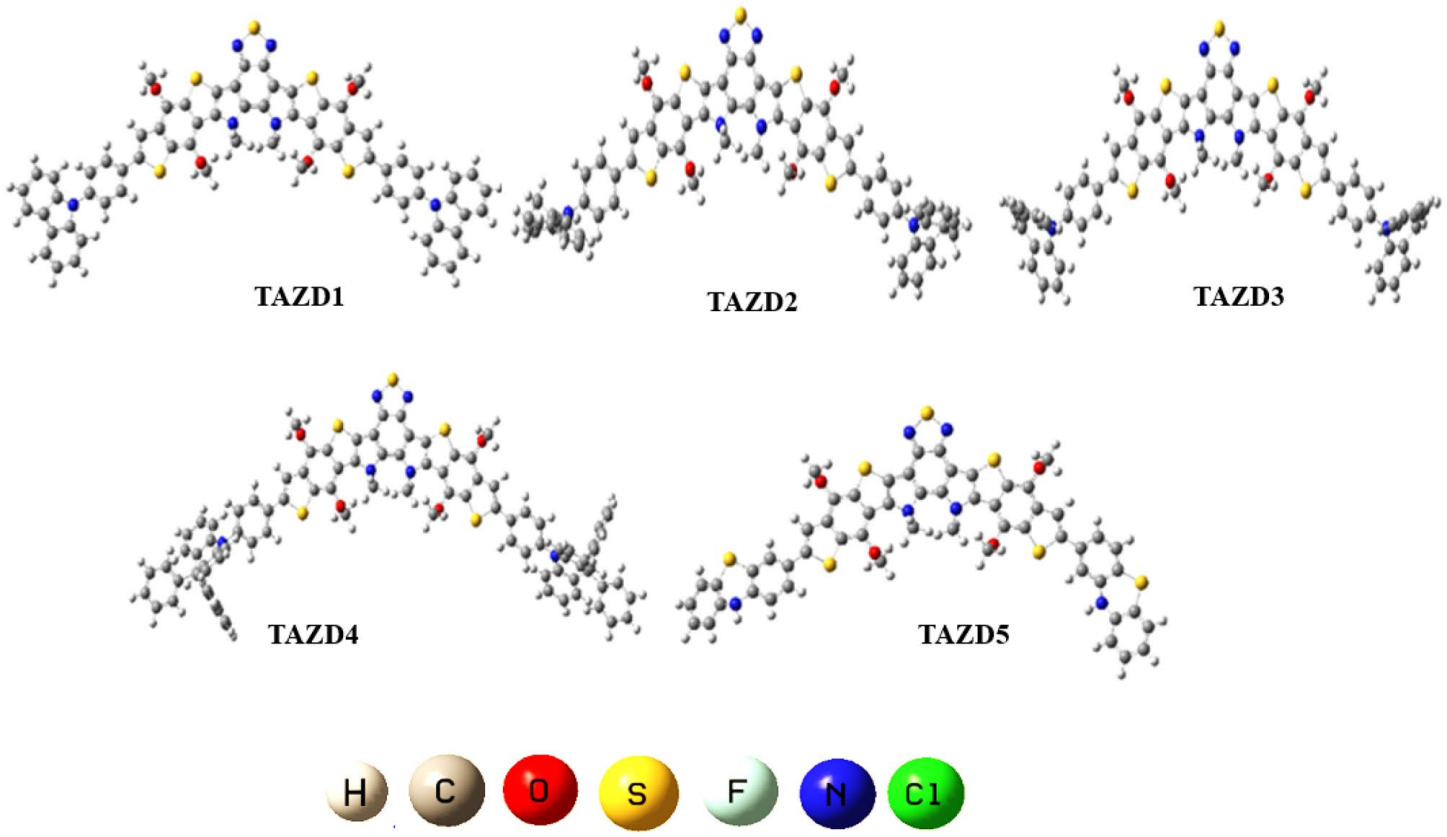
Optimized structures of **TAZD1-TAZD5**. Pictures are created by GaussView 5.0 and Gaussian 09 version D.01 (https://gaussian.com/g09citation/).

In current era, fullerene free-organic systems (FF-OSs) with some special architectures like D-π-A-π-D, A-π-A-π-A^55^, A-D-A^56^ and A-π-A gain significant importance in improving the efficiency of solar cell materials^57–59^. Therefore, in current study we formulated a range of donor nature chromophores (**TAZD1-TAZD5**) with D-π-A-π-D framework from a synthesized system **X94FIC** (A-π-A-π-A)^40^ by molecular replacement at the terminals with efficient azacycle donor moieties (see Fig. 2). First of all, we designed **TAZD1** from **X94FIC** by replacing its terminal acceptors with four rings azacycle donor unit (9-phenyl-9H-carbazole) keeping the central ‘π-linker’ and ‘A’ same as shown in Fig. 1. After that **TAZD2-TAZD5** are designed by replacing the four member azacycle donor rings unit with three, five and six member ring azacycle donor unit as exhibited in Fig. 2. The optimized structures of aforesaid systems are displayed in Fig. 3 while their Chemdraw structures are shown in Fig. S2, however, their IUPAC names are tabulated in Table S1. The utilized azacycle donor moieties: 9-phenyl-9H-carbazole (**THC**), 5-phenyl-10,11-dihydro-5H-dibenzo[b,f]azepine (**THA**), 5-phenyl-5H-dibenzo[b,f]azepine (**TBA**), 9,9,10-triphenyl-9,10-dihydroacridine (**PTH**), 3-methyl-10H-phenothiazine (**MPT**) and their structures can be seen in Fig. S1. We have calculated different parameters like FMOs, DOS, UV–Vis, *V*_*oc*_, E_b_ and TDMs of all the studied compounds. The modifications in derivatives with their respective donor moieties might prove as a significant step towards introducing efficient solar cells.

### Frontier molecular orbitals (FMOs) analysis

**Figure 4:**
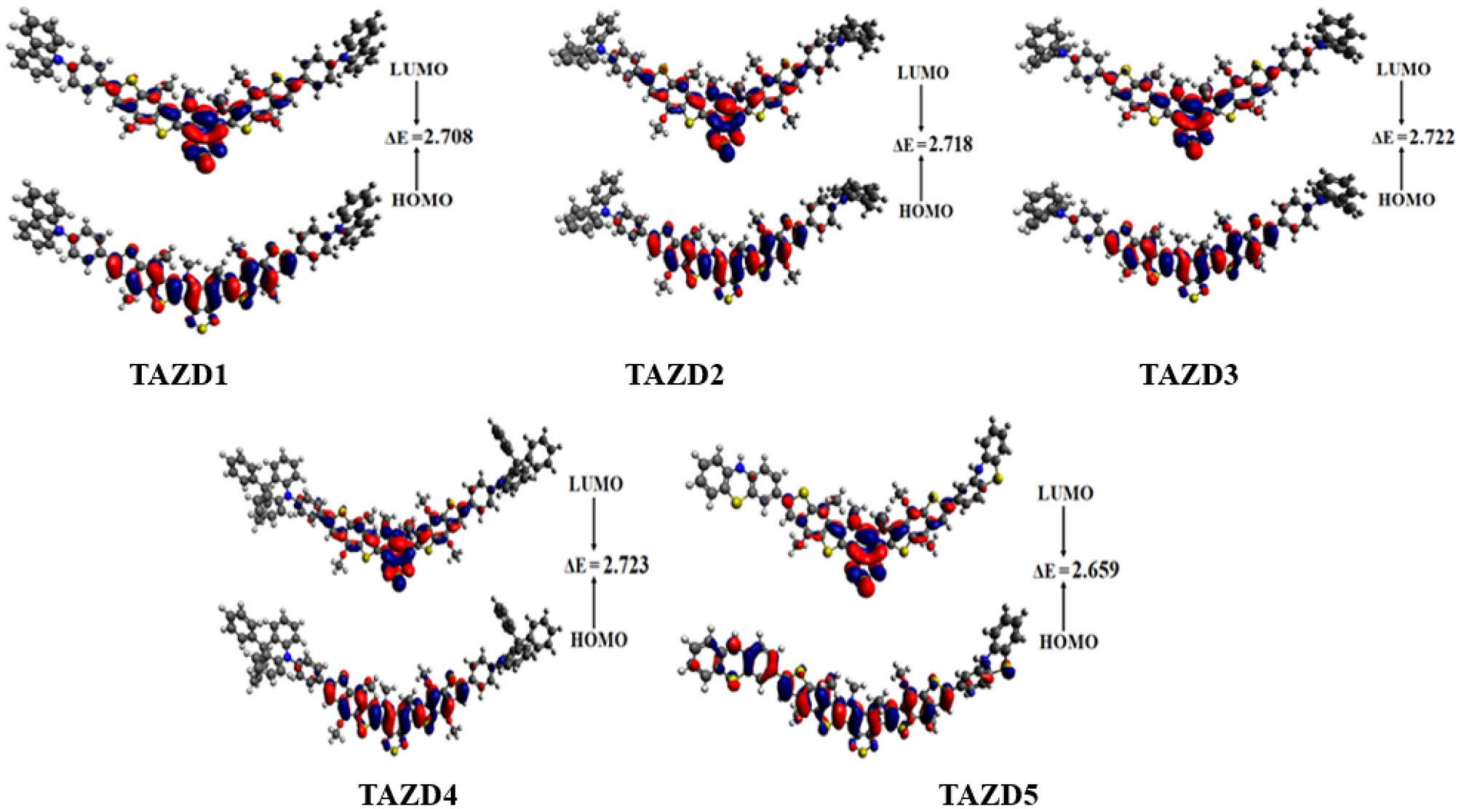
Pictographic representation of HOMOs and LUMOs of **TAZD1-TAZD5**, units are in *eV*. Illustrations are made using Avogadro software, Version 1.2.0. (http://avogadro.cc/).

The optoelectronic properties i.e. charge transfer, electronic features, reactivity, chemical stability and molecular interactions^60^ are investigated via utilizing FMOs^61–63^. The band gap of HOMO/LUMO orbitals is closely linked to these parameters^64^. As HOMO is the electronically filled highest orbital, so it is considered as an electron contributor, whereas, LUMO is considered to be an electron acceptor as it is an empty or unfilled orbital. Molecules having high energy gap (*E*_gap_) values are hard, because they resist changes in electronic configurations, resulting in lower reactivity and increased kinetic stability. Conversely, the compounds with low energy gap are attributed as soft molecules owing to their less stability and higher reactivity. These compounds reveal strong intramolecular charge transfer (ICT) possibilities due to their highly polarized nature and are extremely efficient molecules in the production of solar cell materials^65^. In addition, the HOMO–LUMO band difference is important in calculating a molecule’s total *V*_*oc*_ and *E*_b_^64^. So, FMO analysis is used to compute *E*_HOMO_, *E*_LUMO_ and *E*_gap_ of **TAZD1-TAZD5** and the outcomes are exhibited in Table 1. The pictographs showing charge transference among orbitals are depicted in Fig. 4.

**Table 1:** Computed orbital energies of **TAZD1-TAZD5** and their energy gap.

Compounds	*E*_HOMO_	*E*_LUMO_	$\Delta$E
TAZD1	− 5.177	− 2.469	2.708
TAZD2	− 5.185	− 2.467	2.718
TAZD3	− 5.185	− 2.463	2.722
TAZD4	− 5.199	− 2.476	2.723
TAZD5	− 5.104	− 2.445	2.659

Band gap = *E*_LUMO_ − *E*_HOMO_, units in e*V*.

The above table reveals HOMO energy values for **TAZD1, TAZD2, TAZD3, TAZD4** and **TAZD5** as − 5.177, − 5.185, − 5.185, − 5.199 and − 5.104 eV while energies of LUMO are − 2.469, − 2.467, − 2.463, − 2.476 and − 2.445 eV*,* correspondingly. *E*_gap_ is used to calculate molecules conductivity and net charge transfer^66,67^. The *E*_gap_ values of designed chromophores (**TAZD1-TAZD5**) are revealed as 2.708, 2.718, 2.722, 2.723 and 2.659 eV, correspondingly. Highest energy difference between HOMO and LUMO (2.723 eV) is observed in **TAZD4** among all the other derivatives which may be due to the 9,9-diphenyl-10-(p-tolyl)-9,10-dihydroacridine (**PTH**) donor moiety. The *E*_*gap*_ value is abridged to 2.722 eV in **TAZD3** due to the substitution of **PTH** with 5-(p-tolyl)-5H-dibenzo[b,f]azepine (**TBA**) donor moiety which may be due to the decreased in hindrance in charge transfer of **TBA** as compared to that of **PTH**. Furthermore, the replacement of donor of **TAZD3** i.e. **TBA** with 5-(p-tolyl)-10,11-dihydro-5H-dibenzo[b,f]azepine (**THA**) in **TAZD2** resulted in further reduction of bandgap to 2.718 eV owing to the enhancement in conjugation in the newly introduced donor moiety. **TAZD1** is designed via replacing **THA** with carbazole containing donor moiety i.e. 9-(p-tolyl)-9H-carbazole (**THC**) in which nitrogen atom of carbazole exhibit the electron donating capability. As a result of this, the energy difference is lessen to 2.708 eV in **TAZD1** because of the enhanced push pull mechanism. Moreover, **TAZD5** has exhibited minimum energy gap as compared to all of the studied chromophores owing to the use of phenothiazine-based donor moiety such as 3-methyl-10H-phenothiazine (**MPT**) instead of **THC** in **TAZD1**. The extra electron-rich sulphur atom in phenothiazine might give an improved electron-donating capacity compared to donors that just include nitrogen atoms, like carbazole. Overall, the band gap descending order in the studied compounds is; **TAZD4 > TAZD3 > TAZD2 > TAZD1 > TAZD5**.

The electron density in HOMO of **TAZD1-TAZD4** is predominantly located over the center ‘A’ and ‘π-spacer’ parts of the organic systems and minor over some atoms of donor, while in **TAZD5** the electron density is dispersed on entire system. For LUMO, the electron density is majorly located over π-bridge and core acceptor in **TAZD1-TAZD5**. Among all the investigated compounds, **TAZD5** is found to be the appropriate candidate for future OSCs with enhanced PV behavior due to less energy band gap and adequate charges transition from terminal donors to center acceptor (see Fig. 4).

### UV–Vis analysis

**Figure 5:**
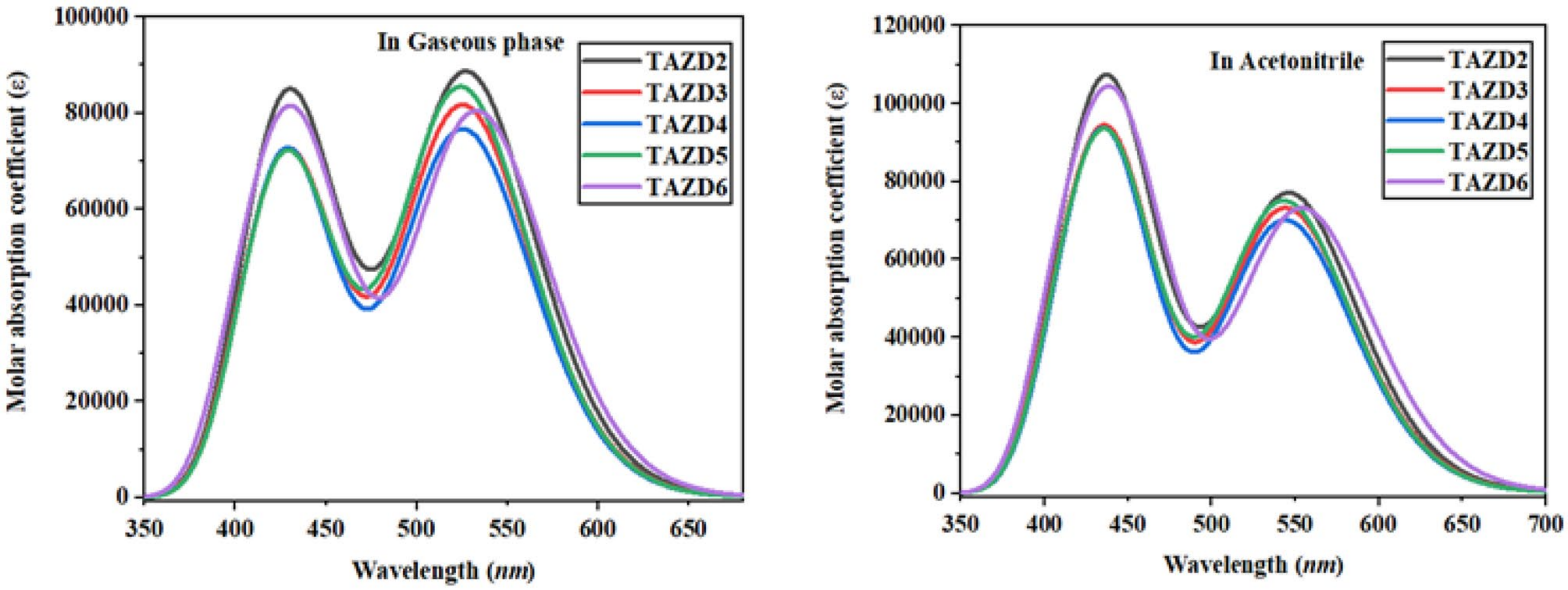
Absorption spectra of **TAZD1-TAZD5** in two different media. The UV–Vis graphs are illustrated utilizing Origin Pro 8.5 version (https://www.originlab.com/).

UV–Vis analysis is significant to investigate the possibility of ICT, kind of configurations of transitions and electronic transitions in a compound. To calculate the absorption spectra of the excited states, the TD-DFT calculations are accomplished in gas and acetonitrile solvent. The observed oscillator strength (*f*_os_), transition energy (*E*), transition type and maximum absorption wavelength (*λ*_max_) are shown in Table 2 as well as Table 3 and other transitions are represented in Tables S2–S11, whereas the absorption spectra of studied compounds **TAZD1-TAZD5** is displayed in Fig. 5.

**Table 2:** Wavelength (*λ*_max_), excitation energy (*E*), oscillator strength (*f*_os_) and nature of molecular orbital contributions of **TAZD1-TAZD5** in acetonitrile.

Compounds	*λ* (*nm*)	E (*eV*)	*f*_os_	MO contributions
TAZD1	546.427	2.269	1.059	H → L (98%)
TAZD2	544.483	2.277	1.006	H → L (98%)
TAZD3	544.101	2.279	0.964	H → L (98%)
TAZD4	543.361	2.282	1.031	H → L (98%)
TAZD5	554.218	2.237	1.005	H → L (96%)

*MO* molecular orbital, *H* HOMO, *L* LUMO.

**Table 3:** Wavelength ($\lambda )$, excitation energy (*E*), oscillator strength (*f*_os_) and nature of molecular orbital contributions of **TAZD1-TAZD5** in gas phase.

Compounds	$\Lambda$ (*nm*)	E (*eV*)	*f*_os_	MO contributions
TAZD1	527.458	2.351	1.216	H → L (97%)
TAZD2	525.579	2.359	1.121	H → L (97%)
TAZD3	525.535	2.359	1.053	H → L (97%)
TAZD4	524.490	2.364	1.173	H → L (97%)
TAZD5	533.219	2.325	1.105	H → L (97%)

In solvent (acetonitrile), all the investigated compounds have revealed maximum absorbance in visible spectrum (Fig. 5). The designed molecules (**TAZD1-TAZD5**) exhibit absorption range from 543.361 to 554.218 *nm* in acetonitrile. In solvent phase, *λ*_max_ values are found to be more red-shifted contrary to gas phase because of solvent effect. Furthermore, the absorption spectra of studied compounds (**TAZD1-TAZD5**) are dominated by π-π interactions^68^. The polar medium results in the stabilization of π-π* state associated with n-π* characteristics by the use of an efficient electronic state^69^. This indicates that, in the stabilization of first singlet state, hydrogen bonding and dipole interactions are imperative^70^ and the molecules exhibit red-shifted absorption as a result of enhancement of solvent polarity.

It is seen that, $\lambda$_max_ values are controlled efficiently by end-capped donor moieties which successively drive the red shifted absorption spectra^71,72^. The absorption band of **TAZD4** is noticed at 543.361 nm having 2.282 eV energy of transition, 1.031 *f*_os_ by exhibiting 98% molecular orbital contribution from HOMO to LUMO. The computed *λ*_max_ value is shifted towards bathochromic shift in **TAZD3** due to the replacement of **PTH** donor of **TAZD4** by **TBA** so, **TAZD3** has exhibited *λ*_max_ at 544.101 nm, 2.279 eV transition energy, and 0.964 oscillator strength via showing HOMO → LUMO MO contribution of 98%. Furthermore, the substitution of **TBA** with **THA** donor moiety resulted in red-shifted absorption of 544.483 nm in **TAZD2** along with lower transition energy (2.277 eV) and 1.006 oscillator strength via same MO contributions. Additionally, **TAZD1** absorption spectra further shifted towards bathochromic shift (546.427 nm), owing to the deposition of another donor moiety *i.e.,* **THC** in **TAZD1** in the replacement of **THA** in **TAZD2**. Finally, the substitution of **TTC** with **MPT** donor moiety resulted in maximum red shift of 554.218 nm in **TAZD5** due to phenothiazine group in **MPT** by showing 96% HOMO → LUMO and 2% HOMO-2 → LUMO molecular orbital contribution at 1.005 oscillator strength and minimum transition energy (2.237 eV) owing to its lowest band gap. $\lambda$_max_ of all the compounds in acetonitrile solvent is found to be in increasing order as **TAZD4** < **TAZD3** < **TAZD2** < **TAZD1** < **TAZD5**.

In gaseous phase (Table 3), all the entitled compounds have almost exhibited equivalent order as well as characteristics as in solvent phase. The absorption spectrum shifts towards the red shift as the dielectric constant of media enhanced. Therefore, greater bathochromic shift is seen in acetonitrile due to its higher dielectric constant than that of gas phase. Nevertheless, it can be concluded from above discussion that, **TAZD5** compound has exhibited maximum absorption wavelength, the lowest transition energy and minimum band gap which implies that, it can be used as an efficient material for photophysical characteristics in non-fullerene OSC materials.

### Open circuit voltage

Open circuit voltage (*V*_oc_) is another significant study that provides insights into the performance of OSCs i.e. their maximum working capability. The total current that can be produced via any optical system can be estimated by *V*_*oc*_^31,55^. So, *V*_oc_ shows direct relation with *E*_HOMO_ and *E*_LUMO_ of donor and acceptor molecules, correspondingly. Thus, *V*_oc_ outcomes of **TAZD1-TAZD5** are determined via Eq. (1) proposed by Scharber and his coworkers.

1 $$V\mathrm{oc}=(\left|{E}_{HOMO}^{D}\right|-\left|{E}_{LUMO}^{A}\right|)-0.3.$$

The major purpose of the calculation of *V*_oc_ is to associate HOMO of the investigated donors with LUMO of PC_61_BM acceptor which is a well-known acceptor having energy of HOMO = − 6.10 eV and energy of LUMO = − 3.70 eV^73^ and the results obtained are represented in Table 4.

**Table 4:** Open circuit voltage and energy driving force of **TAZD1-TAZD5**.

Compounds	*V*_*oc*_*(V)*	$\Delta$ E (*eV*)
TAZD1	1.446	1.746
TAZD2	1.454	1.754
TAZD3	1.454	1.754
TAZD4	1.468	1.768
TAZD5	1.373	1.673

$$\Delta$$***E* = **$${E}_{\mathrm{LUMO}}^{\mathrm{A}}-{E}_{\mathrm{HOMO}}^{\mathrm{D}}$$.

**Figure 6:**
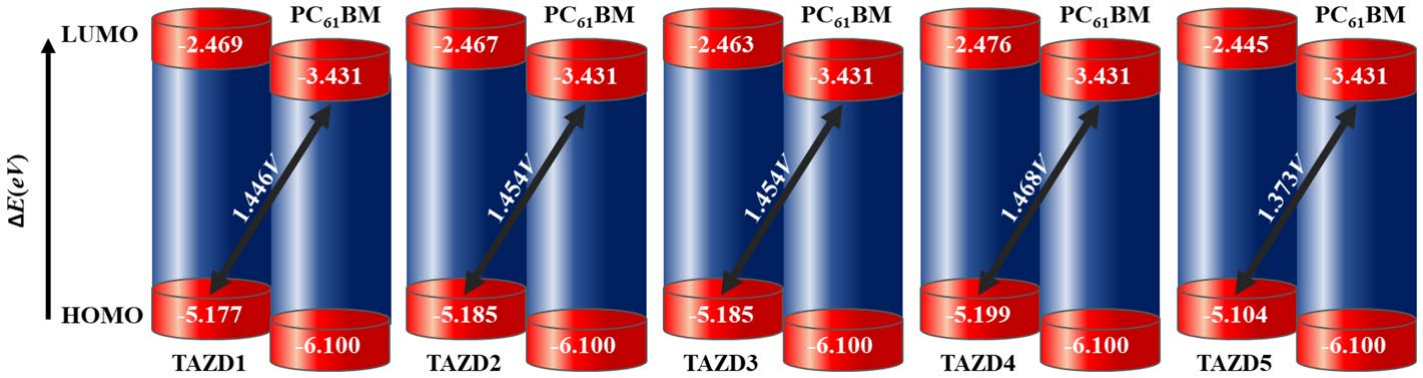
Pictographic representation of *V*_*oc*_ of **TAZD1-TAZD5** with respect to **PC**_**61**_**BM**.

As Table 4 reveals that the values of *V*_*oc*_ for **TAZD1-TAZD5** by considering the energy gap of HOMO_donor_ –LUMO_PC61BM_ are found to be 1.446, 1.454, 1.454, 1.468, and 1.373 V*,* respectively. Among all, **TAZD4** shows maximum results of *V*_oc_. The descending order of open circuit voltage of all the studied molecules is: **TAZD4** > **TAZD2** = **TAZD3** > **TAZD1** > **TAZD5**. From literature, we have found that for a significant transference from D_HOMO_ towards A_LUMO,_ the LUMO of acceptor should be at lesser energy level than that of the LUMO of donor molecules^46,71^. Interestingly, the LUMO of our compounds is higher than the **PC**_**61**_**BM**. These higher values of *V*_*oc*_ elucidate the higher ICT from donor HOMO **TAZD1-TAZD5** towards **PC**_**61**_**BM** which shows highly efficient donating capability of all the studied donors as shown in Fig. 6.

### Density of states (DOS)

**Figure 7:**
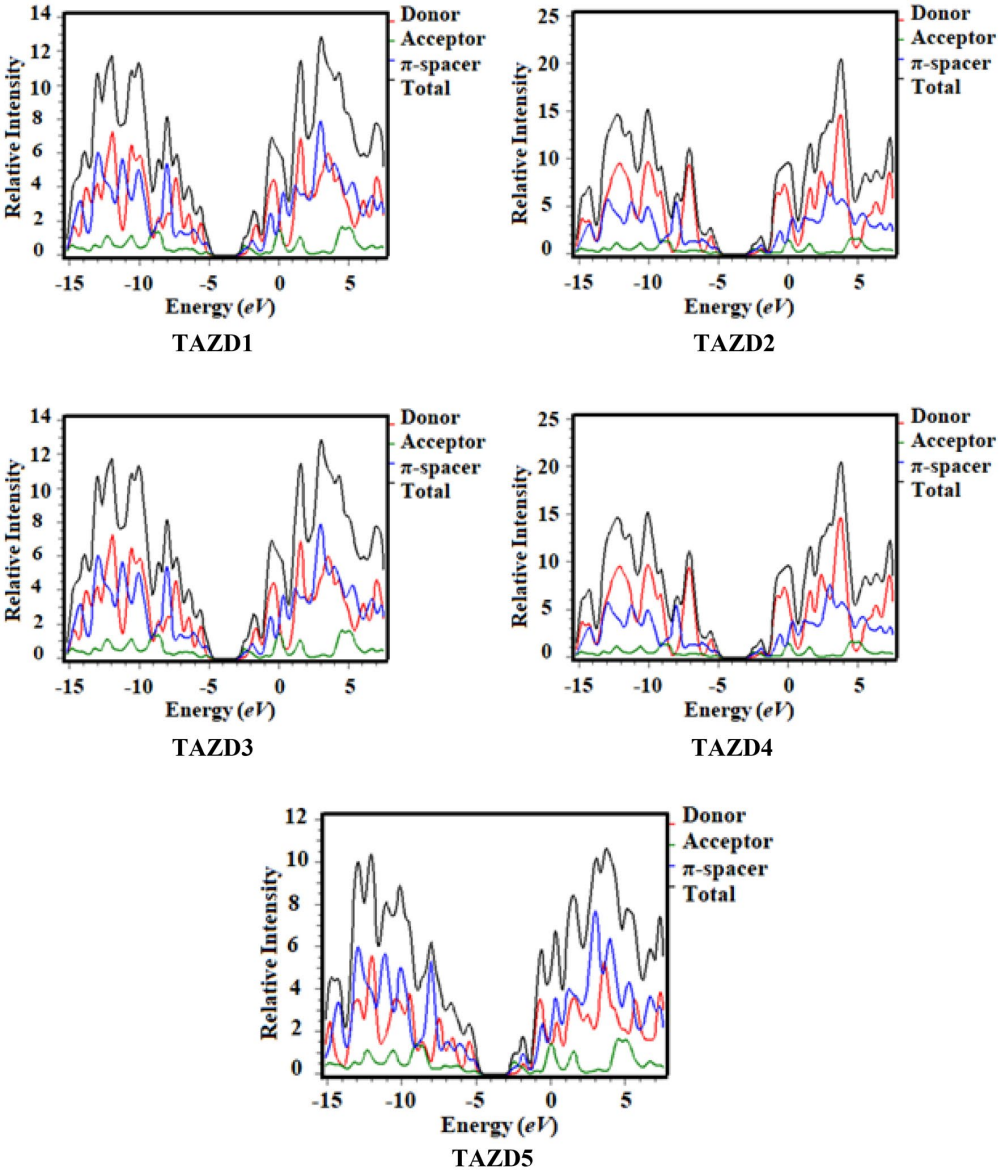
DOS around HOMO and LUMO of **TAZD1-TAZD5**. The DOS pictographs are drawn utilizing PyMOlyze 1.1 version.

DOS was accomplished to assist FMO analysis and their comparative evaluation demonstrates that both of these are analogs to one another. The DOS graphs are displayed in Fig. 7. DOS are performed to disclose the dissemination of electron density on FMOs with the analysis of percentage composition for **TAZD1-TAZD5**. It provides useful data related to contribution of donor as well as acceptor in the development of FMOs. The DOS pictographs illustrate the bonding, non-bonding, and antibonding interactions amongst HOMO and LUMO^74^. The FMO diagrams in the Fig. 7 signify the electronic transitions that demonstrate the intramolecular charge transfer (ICT). In DOS pictographs, the negative values characterize HOMOs while, the positively charged outcomes depict the LUMOs and the difference among their values represents the energy gap on x-axis^75^.

The maximum density on LUMO is noticed at − 2.5 to 4 eV in all the investigated chromophores (**TAZD1-TAZD5**), while on HOMO highest density is observed from − 7 to − 12 eV as shown in Fig. 7. In **TAZD2**, **TAZD4** and **TAZD5** both HOMO and LUMO have comparable charge densities which depict their equal contribution toward FMOs. In **TAZD1-TAZD5**, the donor contributes 7.7, 5.3, 4.8, 4.6 and 22.0% to HOMO, whereas to LUMO its participation is 5.5, 5.5, 5.2, 5.9 and 4.0%, respectively. In the same fashion, π-spacer participates 72.5, 74.4, 74.8, 74.8 and 61.7% to HOMO, while 35.0, 35.2, 34.2, 35.9 and 32.6% to LUMO in **TAZD1-TAZD5**, respectively. Likewise, acceptor shows its percentage participation 19.8, 20.3, 20.4, 20.5 and 16.2% to HOMO in **TAZD1-TAZD5**, respectively, while 59.5, 59.3, 60.6, 58.2 and 63.4% to LUMO, respectively. Overall, the pattern of electronic charge distribution elucidates the delocalization of charges and large amount of charge transfer has taken place in all the modulated chromophores. Interestingly, all the designed derivatives portray almost alike contributions and the electron density is more prominent on the central unit (π-spacer and A units).

### Transition density matrix (TDM) analysis

TDM is considerably utilized for the evaluation of electronic transitions along with their nature for **TAZD1-TAZD5** in solvent phase. The study of the charge carriers localization along with delocalization and the interaction between donor and acceptor groups followed by electron–hole delocalization as calculated by TDM analysis^76^. The role of hydrogen atoms has been neglected because of their small contribution. The TDM heat maps of all the formulated molecules (**TAZD1-TAZD5**) are presented in Fig. S3. To study the transition of electrons within molecules in detail, we split our compound into fragments i.e. donor (D), π-spacer and acceptor (A).

It has been seen that adequate charge is transmitted out of donor towards acceptor moieties as the electron–hole pair is constituted diagonally on the entire TDM plot and represented by clear red and green spots near the acceptor portion. The electron delocalization is seen in the diagonals of A and π-spacers and very little in the D region. Moreover, the charge coherence and electron–hole pair generation are similarly observed in the off-diagonal portions of TDM heat maps (see Fig. S3).

### Hole-electron analysis

**Figure 8:**
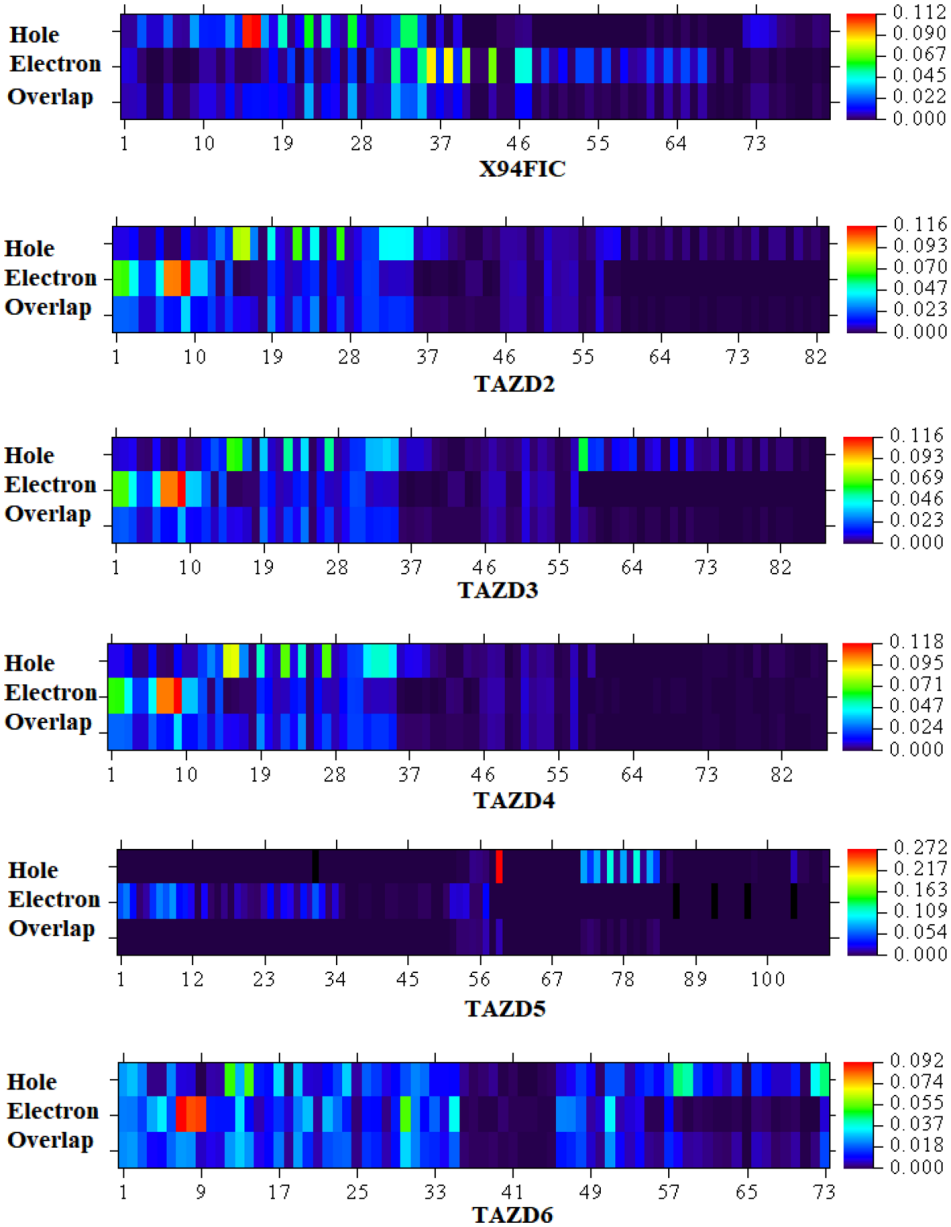
Graphical representation of hole-electron analysis of investigated compounds.

Hole-electron analysis is popularly accomplished by utilizing the Multiwfn 3.8 software. It is a very useful method for revealing the nature of electron excitations. Moreover, it offers a deep understanding of all different electron transfer properties^77, 78^. In this study, hole-electron analysis is performed at B3LYP/6-311G (d,p) to understand the charge transmission in our studied molecules. Figure 8 shows that hole intensity is found maximum at sulphur atom (S15 and S16) of the thiophene ring of π-linker in parent compound (**X94FIC**) while, the hole intensity is observed at C36 and C38 of the acceptor region. Furthermore, it is also clear from Fig. 8 that electron intensity is found at its peak at sulphur atom (S9) of the acceptor region in compounds **TAZD2**, **TAZD3** and **TAZD4** whereas, hole intensity is observed to maximum at sulphur atoms (S15 and S16) of the π-spacer. However, in **TAZD5** hole intensity is higher at methyl group of the π-spacer and electron density is intense at nitrogen atoms (N7 and N8) of the acceptor portion. Moreover, electronic cloud is observed to be thick at nitrogen (N7 and N8) and sulphur (S9) atoms of the acceptor in **TAZD6** however, hole intensity is maximum at carbon atoms (C12 and C14) of the π-linker. The labeled structures of entitled chromophores without hydrogen atoms are illustrated in Fig. S5. In conclusion, all the designed molecules except **TAZD5**, are electron type materials as electronic cloud is observed thick at electronic band in contrast to hole intensity at hole band. In **TAZD5**, hole intensity is found higher at hole band therefore, it is a hole type material.

### Exciton binding energy (E_b_)

One more consideration to estimate the working proficiency, optoelectronic characteristics, and separation potential is binding energy (*E*_b_)^79^. The Eq. (2) is used to compute the binding energy of the studied systems.

2 $$E_{{{\text{b }} = }} E_{{{\text{H}} - {\text{L }} - }} E_{{{\text{opt}}}} .$$

Here, *E*_opt_ represents the least energy that is obtained when an electron moves from S_0_ (ground state) to S_1_ (excited state) during the first electronic transition. *E*_H-L_ signifies the energy difference among HOMO and LUMO whereas, *E*_b_ is the binding energy that is obtained by the difference in band gap between molecular orbitals and first singlet exciton energy. Usually, the lower the value of *E*_b_, greater would be the charge separation and current charge density *J*_*sc*_ which results in higher PCE^80^. The outcomes for the studied compounds calculated in acetonitrile are formulated in Table 5.

**Table 5:** Calculated HOMO–LUMO band gap (*E*_H-L_), first singlet excitation energy (*E*_opt_) and exciton binding energies (*E*_b_) of **TAZD1-TAZD5**.

Compounds	*E*_H–L_(*eV*)	*E*_opt_(*eV*)	*E*_b_(*eV*)
TAZD1	2.708	2.269	0.439
TAZD2	2.718	2.277	0.441
TAZD3	2.722	2.279	0.443
TAZD4	2.723	2.282	0.441
TAZD5	2.659	2.237	0.422

Units in *eV*.

According to our obtained results, the values of *E*_opt_ decreases progressively in all the designed compounds and is found as minimum in **TAZD5** and the same behavior is observed in the HOMO and LUMO energy gap. The binding energy values in **TAZD1-TAZD5** are found to be 0.439, 0.441, 0.443, 0.441 and 0.422 eV*,* correspondingly. The lowermost *E*_b_ value of **TAZD5** depicts that it has excessive charges that can be separated into isolated charges. It is noted that **TAZD5** exhibits high segregation of charges along with high *J*_sc_ which indicates that it is the leading candidate to improve the efficiency of organic photovoltaics. Furthermore, *E*_b_ data unveil a good agreement with TDM outcomes.

### Charge transfer analysis

In order to understand the intermolecular charge transfer between donor and acceptor, a complex is developed between a donor molecule (**TAZD5)** and acceptor (**PC**_**16**_**BM**) polymer and FMO is investigated as shown in Fig. S4. For charge transfer analysis, we selected **TAZD5** due to its unique properties such as reduced band gap and greater UV–Vis absorption spectra etc. among all fabricated chromophores. According to Fig. S4, in HOMO the charge is located over the **TAZD5** donor chromophore and significantly transferred towards the acceptor polymer in LUMO which elucidates the significantly charge transfer from donor towards acceptor.

## Conclusion

In a nutshell, through the molecular engineering with azacycle donor moieties in an organic system** (X94FIC)** fullerene free donor based chromophores **(TAZD1-TAZD5)** were designed. To comprehend their photophysical properties, the behavior of charge transfer, and structure–activity relationship, various analyses were performed at quantum chemical approach. A reasonable energy gap between LUMO/HOMO (∆E = 2.723–2.659 eV) and significant charge transfer with wider absorption spectra (*λ*_max_ = 554.218–543.261 nm in acetonitrile) was examined in all non-fullerene donor chromophores. Additionally, the less binding energy outcomes (E_b_ = 0.422–0.411 eV) in formulated compounds specified higher rate of exciton dissociation that also reinforce the tremendous charge transition out of HOMO towards LUMO as shown by FMOs, DOS and TDMs analyses. Moreover, the *V*_*oc*_ values are also determined with regarding to ${\mathrm{HOMO}}_{\mathrm{donor}}-{\mathrm{LUMO}}_{\mathrm{PC}61\mathrm{BM}}$ and interesting data was found with this order; **TAZD4** (1.199 V) > **TAZD2** (1.185 V) = **TAZD3** (1.185 V) > **TAZD1**(1.177 V) > **TAZD5**(1.104 V). Consequently, significant photovoltaic materials can be developed by structural tailoring with efficient azacycle donor moieties. Moreover. this study also encourages the experimentalist to synthesize these efficient materials for practical use.

### Supplementary Information

Supplementary Information.

## Data Availability

All data generated or analyzed during this study are included in this published article and its supplementary information files**.**
