# Supplementary material for: Influence of azacycle donor moieties on the photovoltaic properties of benzo[c][1,2,5]thiadiazole based organic systems: a DFT study
Source: Sci Rep. 2023 Sep 5;13:14630. doi: 10.1038/s41598-023-41679-0 (PMC10480204; doi:10.1038/s41598-023-41679-0)
Supplement: Supplementary file 1 — Supplementary Information. [file 41598_2023_41679_MOESM1_ESM.docx]

**Supplementary information**

**Influence of Azacycle Donor Moieties on the Photovoltaic Properties of Benzo[*c*][1,2,5]Thiadiazole Based Organic Systems: A DFT Study**

Iqra Shafiq, ^§ a,b^ Muhammad Khalid,* ^§a,b^ Muhammad Adnan Asghar ^c^, Rabia Baby^d^, Ataualpa A. C. Braga,^e^ Saad M. Alshehri,^f^ Sarfraz Ahmed,^g^

^a^Institute of Chemistry, Khwaja Fareed University of Engineering & Information Technology, Rahim Yar Khan, 64200, Pakistan

^b^Centre for Theoretical and Computational Research, Khwaja Fareed University of Engineering & Information Technology, Rahim Yar Khan, 64200, Pakistan

^c^Department of Chemistry, Division of Science and Technology, University of Education Lahore, Pakistan

^d^Department of education, Sukkur IBA university, zip code. 65200

^e^Departamento de Qu´ımica Fundamental, Instituto de Qu´ımica, Universidade de Sao˜ Paulo, Av. Prof. Lineu Prestes, 748, Sao Paulo, 05508-000, Brazil

^f^Department of Chemistry, College of Science, King Saud University, Saudi Arabia

^g^Wellman Center for Photomedicine, Harvard Medical School, Massachusetts General Hospital, Boston, MA 02114, United States

^§^Both authors contributed equally

*Corresponding author's E-mail address:

Muhammad Khalid ([muhammad.khalid@kfueit.edu.pk](mailto:muhammad.khalid@kfueit.edu.pk); [Khalid@iq.usp.br](mailto:Khalid@iq.usp.br))

**Table S1:** The IUPAC names of derivatives **TAZD1-TAZD5**.

| **TAZD1** | 2,12-bis(4-(9H-carbazol-9-yl)phenyl)-4,10,14,17-tetramethoxy-15,16-dimethyl-15,16-dihydro-[1,2,5]thiadiazolo[3,4-e]thieno[2'',3'':4',5']benzo[2',1':4,5]thieno[3,2-b]thieno[3''',2''':4'',5'']benzo[1'',2'':4',5']thieno[2',3':4,5]pyrrolo[3,2-g]indole |
| --- | --- |
| **TAZD2** | 2,12-bis(4-(10,11-dihydro-5H-dibenzo[b,f]azepin-5-yl)phenyl)-4,10,14,17-tetramethoxy-15,16-dimethyl-15,16-dihydro-[1,2,5]thiadiazolo[3,4-e]thieno[2'',3'':4',5']benzo[2',1':4,5]thieno[3,2-b]thieno[3''',2''':4'',5'']benzo[1'',2'':4',5']thieno[2',3':4,5]pyrrolo[3,2-g]indole |
| **TAZD3** | 2,12-bis(4-(5H-dibenzo[b,f]azepin-5-yl)phenyl)-4,10,14,17-tetramethoxy-15,16-dimethyl-15,16-dihydro-[1,2,5]thiadiazolo[3,4-e]thieno[2'',3'':4',5']benzo[2',1':4,5]thieno[3,2-b]thieno[3''',2''':4'',5'']benzo[1'',2'':4',5']thieno[2',3':4,5]pyrrolo[3,2-g]indole |
| **TAZD4** | 2,12-bis(4-(9,9-diphenylacridin-10(9H)-yl)phenyl)-4,10,14,17-tetramethoxy-15,16-dimethyl-15,16-dihydro-[1,2,5]thiadiazolo[3,4-e]thieno[2'',3'':4',5']benzo[2',1':4,5]thieno[3,2-b]thieno[3''',2''':4'',5'']benzo[1'',2'':4',5']thieno[2',3':4,5]pyrrolo[3,2-g]indole |
| **TAZD5** | 4,10,14,17-tetramethoxy-15,16-dimethyl-2,12-di(10H-phenothiazin-3-yl)-15,16-dihydro-[1,2,5]thiadiazolo[3,4-e]thieno[2'',3'':4',5']benzo[2',1':4,5]thieno[3,2-b]thieno[3''',2''':4'',5'']benzo[1'',2'':4',5']thieno[2',3':4,5]pyrrolo[3,2-g]indole |

**Table S2:** Wavelength ($\lambda\mathbf{)}$, excitation energy (*E*), oscillator strength (*f*_os_) and nature of molecular orbital contributions of **TAZD1** in gas phase.

| **NO** | **DFT**  ***λ* (*nm*)** | **E (*eV*)** | ***f*_os_** | **MO contributions** |
| --- | --- | --- | --- | --- |
| 1 | 527.458 | 2.351 | 1.216 | H→L (97%), |
| 2 | 438.587 | 2.827 | 0.745 | H→L+1 (92%), H→L+2 (2%) |
| 3 | 434.012 | 2.857 | 0.076 | H-3→L (17%), H-1→L (71%), H→L+2 (9%) |
| 4 | 414.677 | 2.990 | 0.469 | H-1→L (11%), H→L+2 (83%), H→L+1 (3%) |
| 5 | 404.081 | 3.068 | 0.001 | H-3→L (77%), H-1→L (14%), H-2→L+2 (3%), H→L+2 (2%) |
| 6 | 403.017 | 3.076 | 0.047 | H-2→L (86%), H-4→L (7%), H-3→L+2 (2%) |

**Table S3:** Wavelength ($\lambda\mathbf{)}$, excitation energy (*E*), oscillator strength (*f*_os_) and nature of molecular orbital contributions of **TAZD2** in gas phase.

| **NO** | **DFT**  ***λ* (*nm*)** | **E(*eV*)** | ***f*_os_** | **MO contributions** |
| --- | --- | --- | --- | --- |
| 1 | 525.579 | 2.359 | 1.121 | H→L (97%) |
| 2 | 437.026 | 2.837 | 0.504 | H-2→L (14%), H→L+1 (69%), H-1→L (8%), H-1→L+2 (3%) |
| 3 | 435.506 | 2.847 | 0.073 | H-1→L (72%), H-3→L (6%), H-2→L+2 (6%), H-1→L+1 (4%), H→L+1 (9%) |
| 4 | 434.240 | 2.855 | 0.128 | H-2→L (70%), H→L+1 (17%), H-2→L+1 (3%), H-2→L+2 (2%), H-1→L+2 (5%) |
| 5 | 431.414 | 2.874 | 0.037 | H-3→L (80%), H→L+2 (11%), H-1→L (5%) |
| 6 | 411.361 | 3.014 | 0.397 | H-3→L (11%), H→L+2 (85%) |

**Table S4:** Wavelength ($\lambda\mathbf{)}$, excitation energy (*E*), oscillator strength (*f*_os_) and nature of molecular orbital contributions of **TAZD3** in gas phase.

| **NO** | **DFT**  ***λ* (*nm*)** | **E(*eV*)** | ***f*_os_** | **MO contributions** |
| --- | --- | --- | --- | --- |
| 1 | 525.535 | 2.359 | 1.053 | H→L (97%) |
| 2 | 434.697 | 2.852 | 0.700 | H→L+1 (92%) |
| 3 | 431.760 | 2.872 | 0.063 | H-3→L (83%), H-2→L (3%), H-1→L (2%), H→L+2 (8%) |
| 4 | 429.859 | 2.884 | 0.006 | H-2→L (64%), H-2→L+1 (14%), H-2→L+2 (12%), H-3→L (3%), H-2→L+3 (4%) |
| 5 | 429.621 | 2.886 | 0.004 | H-1→L (65%), H-1→L+2 (19%), H-3→L (2%), H-1→L+1 (6%), H-1→L+3 (3%) |
| 6 | 410.938 | 3.017 | 0.344 | H→L+2 (87%), H-3→L (8%) |

**Table S5:** Wavelength ($\lambda\mathbf{)}$, excitation energy (*E*), oscillator strength (*f*_os_) and nature of molecular orbital contributions of **TAZD4** in gas phase.

| **NO** | **DFT**  ***λ* (*nm*)** | **E(*eV*)** | ***f*_os_** | **MO contributions** |
| --- | --- | --- | --- | --- |
| 1 | 524.490 | 2.364 | 1.173 | H→L (97%) |
| 2 | 453.623 | 2.733 | 0.001 | H-1→L (87%), H-1→L+1 (3%), H-1→L+2 (9%) |
| 3 | 453.357 | 2.735 | 0.001 | H-2→L (87%), H-2→L+1 (5%), H-2→L+2 (7%) |
| 4 | 436.579 | 2.840 | 0.667 | H→L+1 (95%) |
| 5 | 431.610 | 2.873 | 0.044 | H-3→L (85%), H→L+2 (12%) |
| 6 | 412.620 | 3.005 | 0.411 | H-3→L (12%), H→L+2 (84%) |

**Table S6:** Wavelength ($\lambda\mathbf{)}$, excitation energy (*E*), oscillator strength (*f*_os_) and nature of molecular orbital contributions of **TAZD5** in gas phase.

| **NO** | **DFT**  ***λ* (*nm*)** | **E(*eV*)** | ***f*_os_** | **MO contributions** |
| --- | --- | --- | --- | --- |
| 1 | 533.219 | 2.325 | 1.105 | H→L (97%) |
| 2 | 446.468 | 2.777 | 0.097 | H-1→L (88%), H-3→L (7%) |
| 3 | 438.975 | 2.824 | 0.705 | H-2→L (11%), H→L+1 (77%), H→L+2 (5%) |
| 4 | 424.298 | 2.922 | 0.103 | H-2→L (76%), H→L+1 (10%), H-3→L (6%), H→L+2 (4%) |
| 5 | 420.442 | 2.949 | 0.013 | H-3→L (66%), H→L+2 (12%), H-2→L (8%), H-1→L (7%), H→L+1 (2%) |
| 6 | 407.481 | 3.043 | 0.460 | H-3→L (17%), H→L+2 (73%), H→L+1 (5%) |

**Table S7:** Wavelength ($\lambda\mathbf{)}$, excitation energy (*E*), oscillator strength (*f*_os_) and nature of molecular orbital contributions of **TAZD1** in acetonitrile.

| **NO** | **DFT**  ***λ* (*nm*)** | **E(*eV*)** | ***f*_os_** | **MO contributions** |
| --- | --- | --- | --- | --- |
| 1 | 546.427 | 2.269 | 1.059 | H→L (98%) |
| 2 | 445.762 | 2.781 | 1.036 | H→L+1 (96%) |
| 3 | 436.810 | 2.838 | 0.149 | H-3→L (17%), H-1→L (79%), H→L+2 (2%) |
| 4 | 413.998 | 2.995 | 0.569 | H→L+2 (92%), H-1→L (4%) |
| 5 | 401.412 | 3.089 | 0.006 | H-3→L (77%), H-1→L (14%), H→L+2 (3%) |
| 6 | 399.949 | 3.100 | 0.044 | H-4→L (10%), H-2→L (85%) |

**Table S8:** Wavelength ($\lambda\mathbf{)}$, excitation energy (*E*), oscillator strength (*f*_os_) and nature of molecular orbital contributions of **TAZD2** in acetonitrile.

| **NO** | **DFT**  ***λ* (*nm*)** | **E(*eV*)** | ***f*_os_** | **MO contributions** |
| --- | --- | --- | --- | --- |
| 1 | 544.483 | 2.277 | 1.006 | H→L (98%) |
| 2 | 444.101 | 2.792 | 0.933 | H→L+1 (96%) |
| 3 | 435.017 | 2.850 | 0.104 | H-3→L (64%), H-1→L (27%), H-2→L (3%), H→L+2 (2%) |
| 4 | 431.595 | 2.873 | 0.012 | H-3→L (12%), H-2→L (30%), H-1→L (46%), H-1→L+1 (3%), H-1→L+2 (5%) |
| 5 | 430.725 | 2.879 | 0.006 | H-3→L (17%), H-2→L (56%), H-1→L (15%), H-2→L+1 (3%), H-2→L+2 (5%) |
| 6 | 411.771 | 3.011 | 0.520 | H→L+2 (93%), H-3→L (4%) |

**Table S9:** Wavelength ($\lambda\mathbf{)}$, excitation energy (*E*), oscillator strength (*f*_os_) and nature of molecular orbital contributions of **TAZD3** in acetonitrile.

| **NO** | **DFT**  ***λ* (*nm*)** | **E(*eV*)** | ***f*_os_** | **MO contributions** |
| --- | --- | --- | --- | --- |
| 1 | 544.101 | 2.279 | 0.964 | H→L (98%) |
| 2 | 442.295 | 2.803 | 0.938 | H→L+1 (95%) |
| 3 | 434.027 | 2.857 | 0.117 | H-3→L (90%), H-1→L (2%), H→L+2 (3%) |
| 4 | 431.234 | 2.875 | 0.002 | H-2→L (70%), H-2→L+2 (12%), H-2→L+1 (9%), H-2→L+3 (3%) |
| 5 | 430.845 | 2.878 | 0.002 | H-1→L (71%), H-1→L+2 (13%), H-3→L (2%), H-1→L+1 (8%), H-1→L+3 (3%) |
| 6 | 412.045 | 3.009 | 0.457 | H→L+2 (93%), H-3→L (3%) |

**Table S10:** Wavelength ($\lambda\mathbf{)}$, excitation energy (*E*), oscillator strength (*f*_os_) and nature of molecular orbital contributions of **TAZD4** in acetonitrile.

| **NO** | **DFT**  ***λ* (*nm*)** | **E(*eV*)** | ***f*_os_** | **MO contributions** |
| --- | --- | --- | --- | --- |
| 1 | 543.361 | 2.282 | 1.031 | H→L (98%), |
| 2 | 444.452 | 2.790 | 0.912 | H→L+1 (96%), |
| 3 | 437.813 | 2.832 | 0.004 | H-1→L (87%), H-1→L+1 (3%), H-1→L+2 (5%) |
| 4 | 437.473 | 2.834 | 0.003 | H-2→L (87%), H-2→L+1 (4%), H-2→L+2 (5%), H-1→L (2%) |
| 5 | 433.602 | 2.859 | 0.104 | H-3→L (92%), H→L+2 (4%) |
| 6 | 413.060 | 3.002 | 0.527 | H→L+2 (93%), H-3→L (4%) |

**Table S11:** Wavelength ($\lambda\mathbf{)}$, excitation energy (*E*), oscillator strength (*f*_os_) and nature of molecular orbital contributions of **TAZD5** in acetonitrile.

| **NO** | **DFT**  ***λ* (*nm*)** | **E(*eV*)** | ***f*_os_** | **MO contributions** |
| --- | --- | --- | --- | --- |
| 1 | 554.218 | 2.237 | 1.005 | H→L (96%), H-2→L (2%) |
| 2 | 461.594 | 2.686 | 0.108 | H-1→L (93%), H-3→L (4%) |
| 3 | 449.740 | 2.757 | 0.734 | H-2→L (29%), H→L+1 (62%), H-1→L+2 (2%) |
| 4 | 438.262 | 2.829 | 0.379 | H-2→L (65%), H→L+1 (30%), |
| 5 | 425.595 | 2.913 | 0.011 | H-3→L (82%), H→L+2 (10%), H-1→L (3%), H-1→L+1 (3%) |
| 6 | 411.225 | 3.015 | 0.640 | H-3→L (12%), H→L+2 (74%), H-1→L+1 (8%) |

|   9-phenyl-9H-carbazole (**THC**), |   5-phenyl-10,11-dihydro-5H-dibenzo[b,f]azepine (**THA**) |
| --- | --- |
|   5-phenyl-5H-dibenzo[b,f]azepine (**TBA**) |   9,9,10-triphenyl-9,10-dihydroacridine (**PTH)** |
|   3-methyl-10H-phenothiazine (**MPT**)  **Figure S1:** The 2D display of donor moieties used in**TAZD1-TAZD5** | |


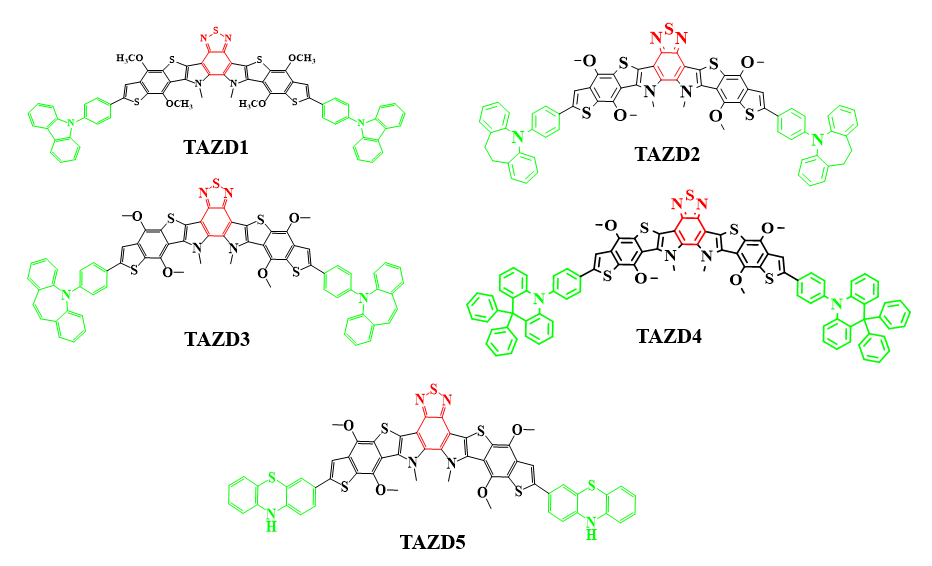


**
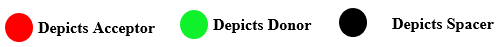
**

**Figure S2:** The 2D display of **TAZD1-TAZD5**.

| 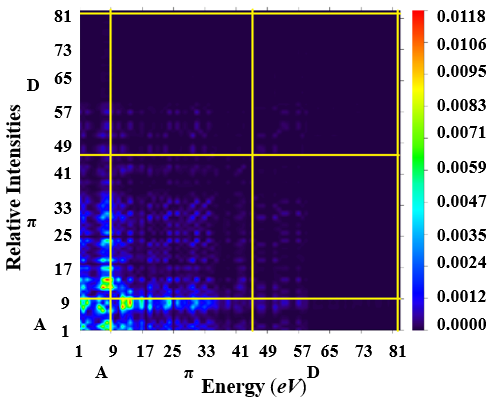 | 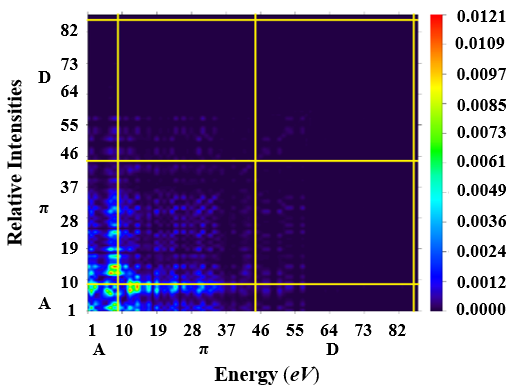 |
| --- | --- |
| **TAZD1** | **TAZD2** |
| **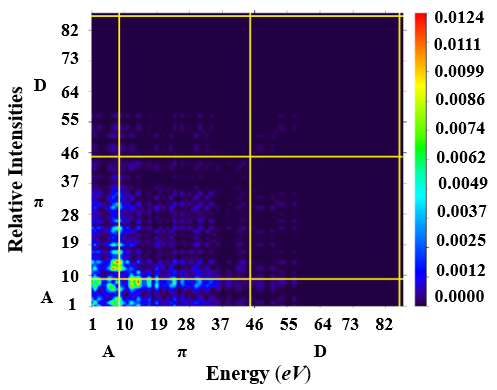** | **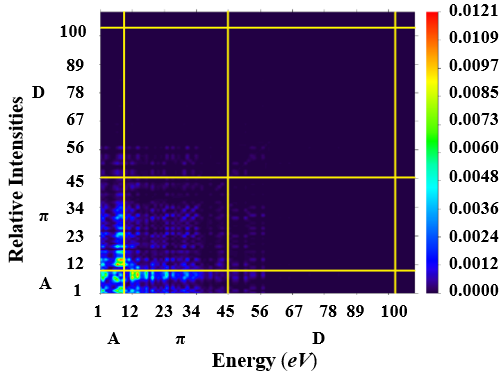** |
| **TAZD3** | **TAZD4** |
| **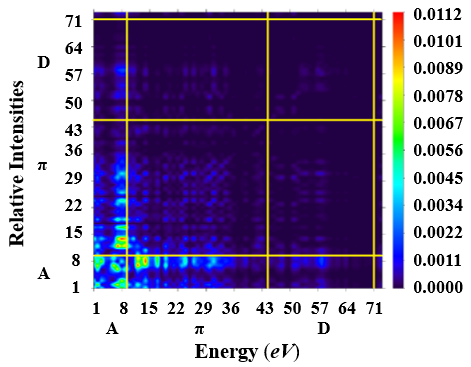** | |
| **TAZD5** | |

**Figure S3:** TDM heat maps for **TAZD1-TAZD5**


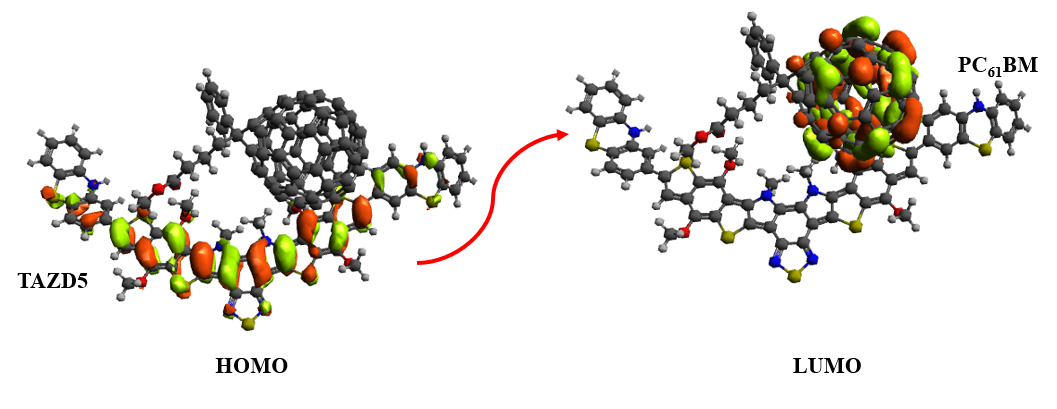


**Figure S4**: Intermolecular charge transfer molecular analysis between TAZD5 and PC_61_BM


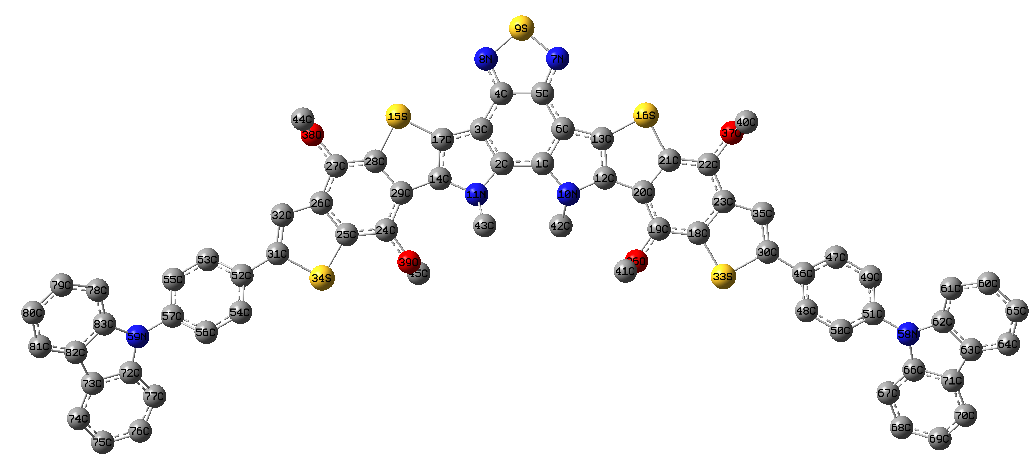


**TAZD1**

**
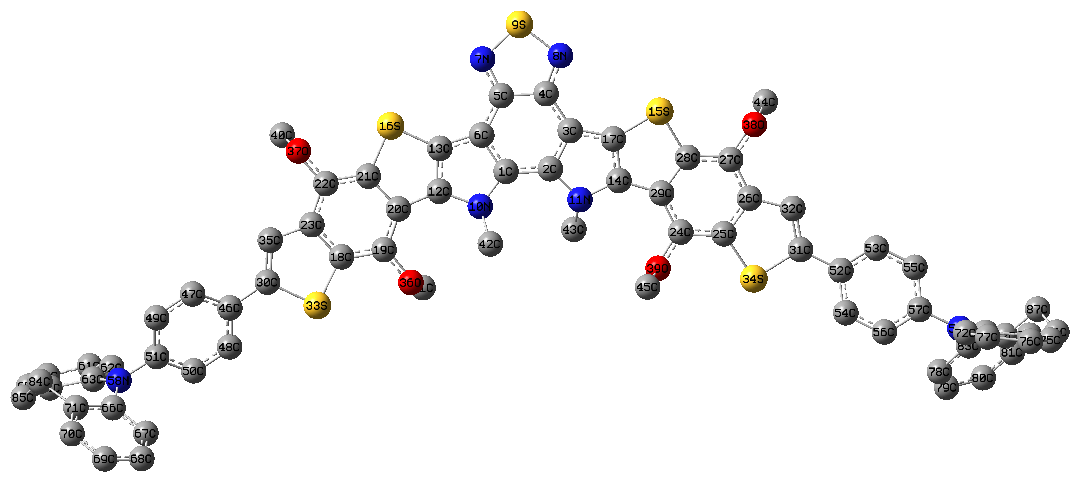
**

**TAZD2**

**
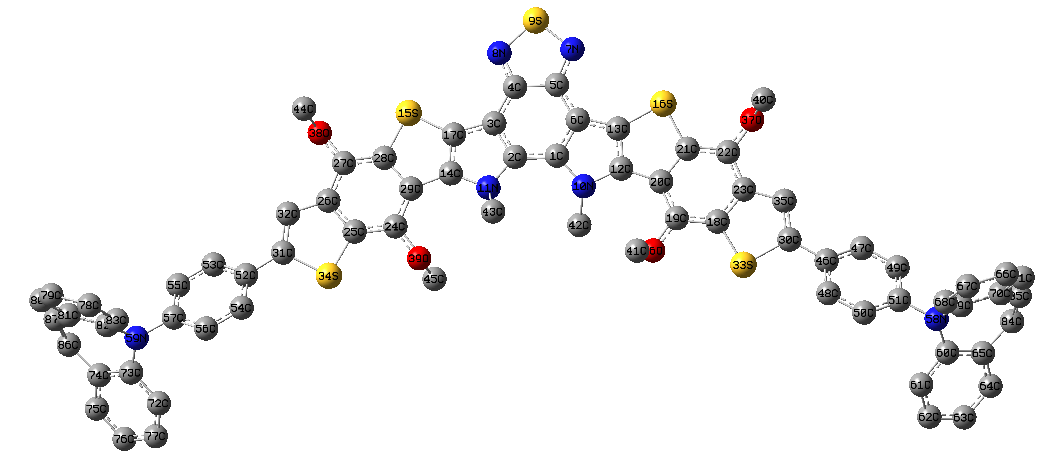
**

**TAZD3**

**
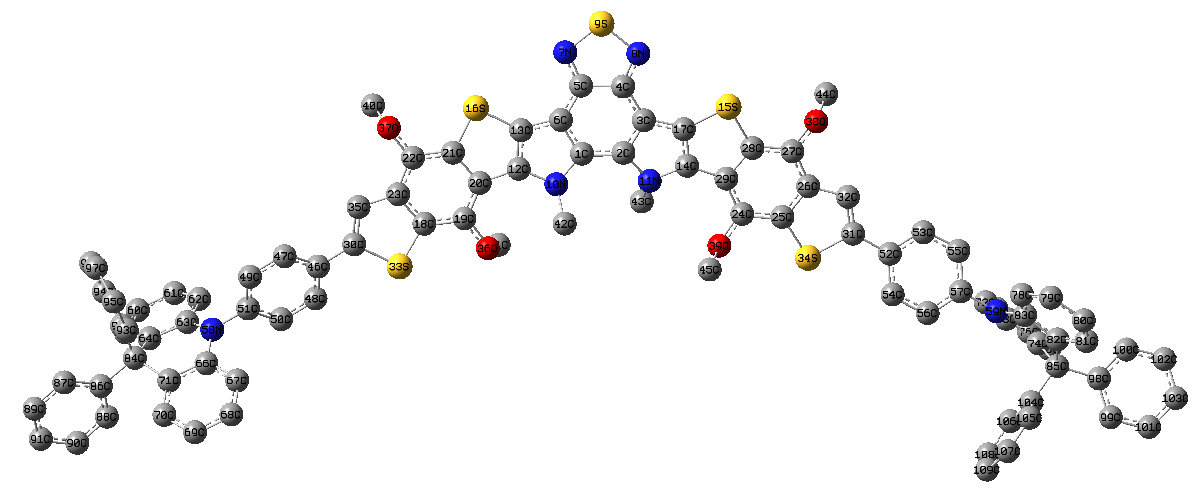
**

**TAZD4**

**
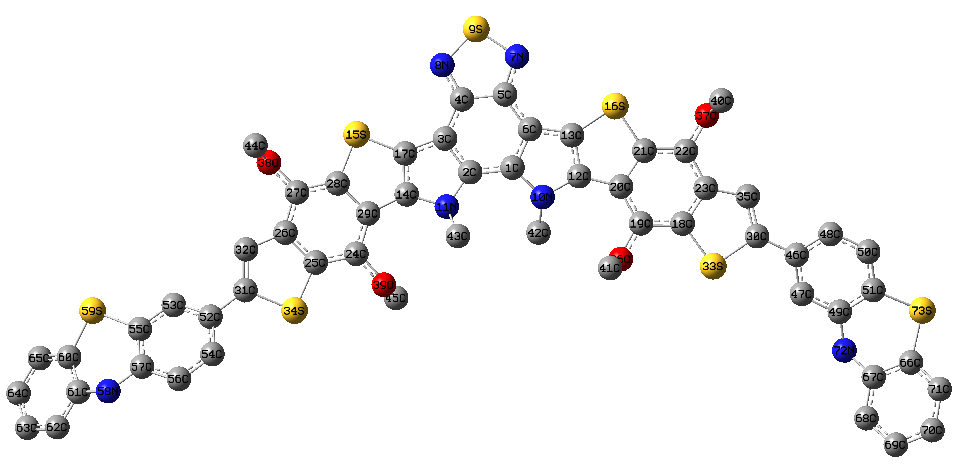
**

**TAZD5**

**Figure S5.** The labeled structures of entitled chromophores without hydrogen atoms
